# A Fully Integrated Real-Time Detection, Diagnosis, and Control of Community Diarrheal Disease Clusters and Outbreaks (the INTEGRATE Project): Protocol for an Enhanced Surveillance System

**DOI:** 10.2196/13941

**Published:** 2019-09-26

**Authors:** Kirsty Marie McIntyre, Frederick J Bolton, Rob M Christley, Paul Cleary, Elizabeth Deja, Ann E Durie, Peter J Diggle, Dyfrig A Hughes, Simon de Lusignan, Lois Orton, Alan D Radford, Alex J Elliot, Gillian E Smith, Darlene A Snape, Debbi Stanistreet, Roberto Vivancos, Craig Winstanley, Sarah J O’Brien

**Affiliations:** 1 Department of Epidemiology and Population Health Institute of Infection and Global Health University of Liverpool Neston United Kingdom; 2 National Institute for Health Research Health Protection Research Unit in Gastrointestinal Infections Liverpool United Kingdom; 3 Field Epidemiology Services Public Health England Liverpool United Kingdom; 4 Department of Public Health and Policy Institute of Psychology, Health and Society University of Liverpool Liverpool United Kingdom; 5 Centre for Health Informatics, Computing, and Statistics Lancaster Medical School Lancaster University Lancaster United Kingdom; 6 Centre for Health Economics and Medicines Evaluation Bangor University Bangor United Kingdom; 7 Royal College of General Practitioners Research and Surveillance Centre London United Kingdom; 8 Nuffield Department of Primary Care Health Sciences University of Oxford Oxford United Kingdom; 9 Department of Infection Biology Institute of Infection and Global Health University of Liverpool Neston United Kingdom; 10 Real-Time Syndromic Surveillance Team Field Service, National Infection Service Public Health England Birmingham United Kingdom; 11 Department of Clinical Infection, Microbiology and Immunology Institute of Infection and Global Health University of Liverpool Liverpool United Kingdom

**Keywords:** gastrointestinal diseases, syndromic surveillance, microbiology, diarrhea, vomiting

## Abstract

**Background:**

Diarrheal disease, which affects 1 in 4 people in the United Kingdom annually, is the most common cause of outbreaks in community and health care settings. Traditional surveillance methods tend to detect point-source outbreaks of diarrhea and vomiting; they are less effective at identifying low-level and intermittent food supply contamination. Furthermore, it can take up to 9 weeks for infections to be confirmed, reducing slow-burn outbreak recognition, potentially impacting hundreds or thousands of people over wide geographical areas. There is a need to address fundamental problems in traditional diarrheal disease surveillance because of underreporting and subsequent unconfirmed infection by patients and general practitioners (GPs); varying submission practices and selective testing of samples in laboratories; limitations in traditional microbiological diagnostics, meaning that the timeliness of sample testing and etiology of most cases remains unknown; and poorly integrated human and animal surveillance systems, meaning that identification of zoonoses is delayed or missed.

**Objective:**

This study aims to detect anomalous patterns in the incidence of gastrointestinal disease in the (human) community; to target sampling; to test traditional diagnostic methods against rapid, modern, and sensitive molecular and genomic microbiology methods that identify and characterize responsible pathogens rapidly and more completely; and to determine the cost-effectiveness of rapid, modern, sensitive molecular and genomic microbiology methods.

**Methods:**

Syndromic surveillance will be used to aid identification of anomalous patterns in microbiological events based on temporal associations, demographic similarities among patients and animals, and changes in trends in acute gastroenteritis cases using a point process statistical model. Stool samples will be obtained from patients’ consulting GPs, to improve the timeliness of cluster detection and characterize the pathogens responsible, allowing health protection professionals to investigate and control outbreaks quickly, limiting their size and impact. The cost-effectiveness of the proposed system will be examined using formal cost-utility analysis to inform decisions on national implementation.

**Results:**

The project commenced on April 1, 2013. Favorable approval was obtained from the Research Ethics Committee on June 15, 2015, and the first patient was recruited on October 13, 2015, with 1407 patients recruited and samples processed using traditional laboratory techniques as of March 2017.

**Conclusions:**

The overall aim of this study is to create a new One Health paradigm for detecting and investigating diarrhea and vomiting in the community in near-real time, shifting from passive human surveillance and management of laboratory-confirmed infection toward an integrated, interdisciplinary enhanced surveillance system including management of people with symptoms.

**International Registered Report Identifier (IRRID):**

DERR1-10.2196/13941

## Introduction

### Background

Diarrheal disease, affecting 1 in 4 people in the United Kingdom annually [1], is the most common cause of infectious disease outbreaks in community and health care settings. Traditional surveillance methods tend to detect point-source outbreaks of diarrhea and vomiting; however, they are less effective at identifying low-level and intermittent contamination of the food supply, unless the organism is very rare. Furthermore, it may take up to 9 weeks for infections to be confirmed by a reference laboratory, reducing recognition of *slow-burn* outbreaks that can affect hundreds or thousands of people over a wide geographical area.

There is a need to address fundamental problems inherent in traditional surveillance for diarrheal disease. First, surveillance depends on the examination of stool samples obtained from symptomatic patients attending their general practitioner (GP). The submission practices and selective testing of samples in laboratories can vary and potentially fragment current laboratory-based surveillance systems. Furthermore, as fewer people present in person to their GP, laboratory-based systems have become less sensitive; the *hidden* burden of disease has increased [1]. Second, limitations of traditional microbiological diagnostic methods mean that the etiology of diarrhea in most cases remains unknown. Third, diagnostics are conducted in a hierarchical manner (local detection, confirmation, and typing centrally at national reference laboratories), which can take several days and can be delayed during busy periods such as when an outbreak investigation is underway. Finally, although many diarrheal diseases are zoonotic, human and animal surveillance systems are poorly integrated, meaning that identification of zoonotic events, including emergence of new or antibiotic-resistant strains, is delayed or missed altogether.

### Overall Study Aim

The overall aim of this study is to create a new *One Health* paradigm for detecting and investigating diarrhea and vomiting in the community in near-real time, shifting from passive human surveillance for gastrointestinal (GI) illness and management of laboratory-confirmed infection toward an integrated, interdisciplinary enhanced surveillance system including management of people with symptoms.

### Contribution to the Field

The comparison of the use of syndromic surveillance for cluster detection and targeted sampling within the community with the use of traditional surveillance will provide a series of improvements to the surveillance of GI disease. We hypothesize that enhanced GI surveillance will allow the following:

- Faster identification of outbreaks of GI disease
- More accurate characterization of the *hidden* burden of disease (underreporting of episodes of illness in which patients do not visit GPs in person). This will result in an observed increase in the incidence of outbreaks
- Identification of a greater number of routes for transmission of pathogens that cause GI illness.

The integration of human and animal syndromic surveillance systems and the use of modern microbiological methods within this project are hypothesized to facilitate (1) faster detection of zoonotic transmission events; (2) earlier identification of a greater spectrum of disease-transmitting pathogens, reducing the diagnostic gap for GI disease; and (3) a reduction in the numbers of false-positive and false-negative stool samples.

There will be differences in the costs and benefits of using improved surveillance methods to detect outbreaks of GI disease earlier compared with using traditional surveillance methods. Potentially, these differences could be in parameters relating to host-pathogen interactions; rate parameters that define the transition of patients among relevant states (eg, susceptible, diseased, and symptomatic and/or infectious); test characteristics, defined by sensitivity, specificity, and positive and negative predictive values; costs (associated with screening, patients’ use of National Health Service [NHS] community, primary and secondary care services, treatments, and other investigations); health outcomes (defined by health state utilities); personal social services; days absent from work or education; and other potential cost impacts.

### Overall Objectives of the Research Program

The overall objectives of this research program are to (1) develop and implement new sampling and microbiological testing algorithms, including strategies for pathogen discovery and evolutionary biology; (2) run the new system alongside the existing system to assess its performance against a set of outcome-based indicators including time to detection of event, compliance with sampling among people with symptoms, numbers of false-positive and false-negative stool samples, and diagnostic yield; and (3) determine the costs and benefits of the new system.

## Methods

### Setting

The setting is the North West area of England (population 7.1 million).

### Case Recruitment and Informed Consent

A total of 4 data streams will feed in real time into the new surveillance program. They are NHS 111 telephone triage data on symptoms of vomiting and diarrhea (a real-time syndromic surveillance system operated by Public Health England [PHE]), data from the Small Animal Veterinary Surveillance Network [2], and *Salmonella* data from the Animal and Plant Health Agency (APHA).

**Figure 1:**
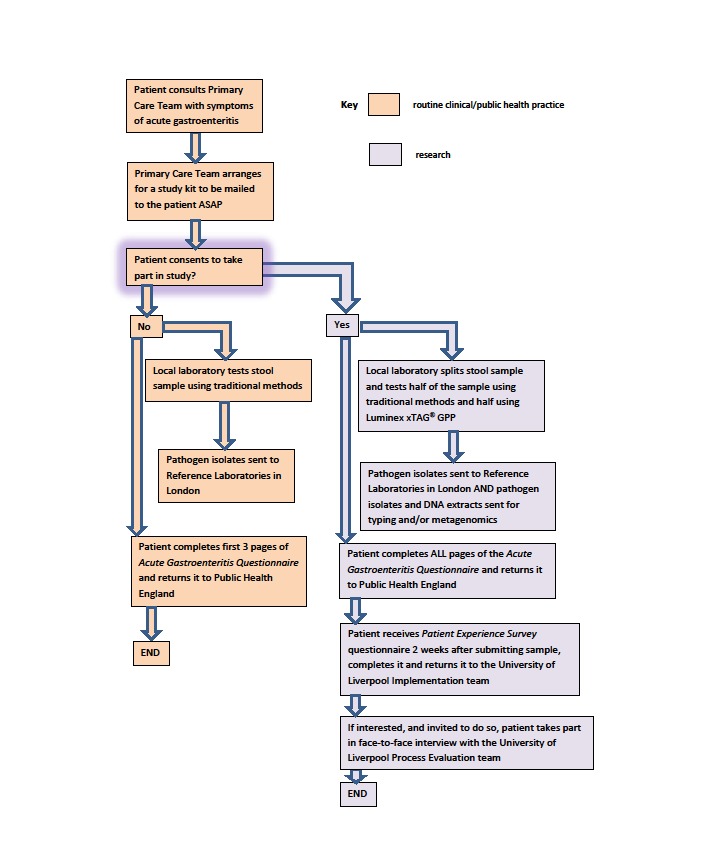
Patient recruitment flow diagram and study processes for the INTEGRATE project. ASAP: as soon as possible; xTAG GPP: Luminex xTAG Gastrointestinal Pathogen Panel.

**Figure 2:**
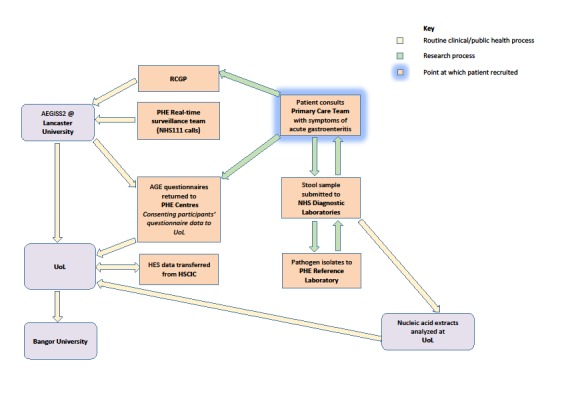
Post patient recruitment data flow for the INTEGRATE project. AEGISS: Ascertainment and Enhancement of Gastrointestinal Surveillance and Statistics; AGE: Public Health Acute Gastroenteritis; HES: Hospital Episode Statistics; HSCIC: Health and Social Care Information Centre; NHS: National Health Service; PHE: Public Health England; RCGP: Royal College of General Practitioners; UoL: University of Liverpool.

The fourth data stream will be derived from general practices in the Royal College of General Practitioners’ Research and Surveillance Centre National Monitoring Network (RCGP RSC NMN) [3]. Members of the public with symptoms of acute gastroenteritis including a case definition of vomiting and diarrhea who seek health advice from general practices in the RCGP RSC NMN will be invited to submit a stool sample for microbiological examination. Their consent for this procedure will be sought because normal care would not necessarily entail stool sampling for most patients unless their symptoms were severe or had persisted for a long time. It is possible that most patients will be recruited as part of a telephone consultation with a member of their primary health care team (physician assessment by telephone). The primary health care team will arrange for the patient to receive through the post an invitation letter, an information sheet about the study, a consent form, a stool sampling kit with a reply-paid envelope, and a short Public Health Acute Gastroenteritis questionnaire (which is part of routine public health practice) with a reply-paid envelope. Patients who present at a general practice in the RCGP RSC NMN will receive these items in person. Patients who consent to take part and provide a stool sample will be recruited into the study. Consent statements agreed to in the study consent form include acknowledgment that taking part in the study is voluntary and that consenting patients can leave at any time. Figures 1 and 2 describe the study recruitment procedure, processes, and data flows using flow diagrams.

We aim to recruit 6000 participants. This will allow us to detect the period (annual) prevalence of symptomatic GI infection in the community of 20% ± 1%.

### Sample Processing

On receipt at 1 of the 3 diagnostic laboratories taking part (Royal Liverpool and Broadgreen University Hospitals NHS Trust, Central Manchester University Hospitals NHS Foundation Trust, or Lancashire Teaching Hospitals NHS Foundation Trust), the stool sample will be divided into 2 parts. One part of the sample will be processed according to routine clinical practice at each of the 3 laboratories. The other half will be processed with a rapid first-line diagnostic screen, using a molecular multiplex real-time polymerase chain reaction (PCR) assay (a commercially available CE-marked kit [Luminex xTAG Gastrointestinal Pathogen Panel, xTAG GPP] [4]), which incorporates most of the major community GI pathogens relevant to the United Kingdom. This will be complemented by tests for Enteroaggregative *Escherichia coli* and Sapovirus, which have been incorporated into the xTAG GPP assay, using assays already developed for PHE’s Olympics Response [5]. Downstream from the rapid xTAG GPP diagnosis, an algorithm for testing stool samples from presumed outbreaks using next-generation sequencing technologies will allow for molecular characterization of known pathogens. Where a pathogen has not been identified (likely in 60% of samples), samples from clinically severe outbreaks will be fast-tracked and characterized using a relatively low-throughput platform for combined RNA/DNA viromes and bacterial metagenomes. If they are from less clinically urgent cases, they will be sequenced at reduced cost on a high-throughput platform.

If a patient does not wish to take part in the study but does submit a stool sample, this case will be processed according to routine clinical practice.

All results will be reported to the patient’s GP. Results from the Luminex assays will be issued as an interim report through *Telepath*, which is the routine electronic reporting system between diagnostic laboratories and general practice, and the results of the routine clinical practice assays will be issued as a final report through *Telepath*. In each laboratory, an experienced consultant medical microbiologist, who is a coinvestigator on the grant with research time costed into it, will be available to discuss any discrepant results with the patient’s GP. The most likely scenario is that a stool sample that provides negative results using traditional methods will provide positive results using the xTAG GPP. This is to be expected because the xTAG GPP, which is a multiplexed PCR, will detect the presence of pathogen DNA/RNA in stool even if an organism has died-off in the sample during transit to the laboratory.

### Public Health Acute Gastroenteritis Questionnaires

Public Health Acute Gastroenteritis (AGE) questionnaires will be returned to PHE's health protection teams using reply-paid envelopes. These questionnaires contain the routine follow-up information that is collected and collated by the PHE to assist outbreak detection or investigation. In addition, these questionnaires will contain questions on quality of life during the acute illness. Quality of life will be measured using the EuroQol-5D-3L [6], which is the gold standard tool. This gives snapshots of quality of life at points in time. At this time point (time point 1), we will capture the quality of life during the acute illness.

### Resource Use and Costs

Resource use and costs will be assessed from several different perspectives, described below, including the public sector and patients.

#### Public Sector, Including National Health Service and Social Services

Consistent with National Institute for Health and Care Excellence’s (NICE) methods for the development of public health guidance, we will adopt a public sector costing perspective. Although productivity costs are not routinely included in analyses, we will collect the data for consideration in sensitivity analysis. Unit costs will be derived from standard sources such as the Personal Social Services Research Unit Costs of Health and Social Care, the British National Formulary for drug costs, and NHS reference costs. The costs of laboratory and public health services will be obtained from PHE.

#### Patients

Patients who indicate in their consent form that they are willing to be contacted about a Patient Experience Survey (PES) will receive a questionnaire that seeks information about resource use 2 weeks after returning their stool sample pot. Most patients will have recovered fully 2 weeks later, and therefore, a complete picture of the costs they incurred because of their illness should be available. The short resource use questionnaires will capture details about the use of health care services, personal social services, days absent from work or school, and other potential costs. Quality of life, using EuroQol-5D-3L, will be assessed during the acute episode (time point 1) and at 2 weeks post recovery (time point 2). It will be used to calculate quality-adjusted life-years (QALYs).

#### Hospital Episode Statistics

Patients’ use of secondary care services will be assessed by accessing Hospital Episode Statistics data sourced from NHS Digital. This will require a bespoke download, matching patients who have consented according to their NHS number, name, and date of birth, following a standard operating procedure to ensure patient anonymity and data protection.

#### Costs of Technology

These will include setup costs, including capital costs, the marginal costs of delivering the service, and the cost implications for patients using the NHS services. These costs will be obtained from Luminex and from time and motion studies conducted at participating microbiology laboratories.

#### Patient Experience Survey

The purpose of this survey is to explore service users’ and, where relevant, their caregivers’ perceptions and experiences of accessing the INTEGRATE service. Questions contained within the PES were developed from themes identified from the extant literature and include (1) motivations for accessing health care in relation to diarrhea; (2) predisposing or enabling motivations for accessing a given health care service; (3) experience and perceptions including the acceptability of self-stool sampling; and (4) dimensions of patient satisfaction, including, for example, communication and clarity of information.

Members of the public eligible to participate in the survey will be either a patient (aged ≥16 years) who has recently suffered from diarrhea and provided a stool sample, or a person who has parental, guardian, or caregiver responsibility for, and who will act as a proxy on behalf of, a patient who has recently suffered from diarrhea and provided a stool sample.

There was no upper age limit for sampling patients. However, where the patient is a minor (aged <16 years), the parent, guardian, or caregiver will be invited to respond on their behalf (as a proxy). We estimate approximately 5000 members of the public will be eligible to take part in the survey within a time frame of 15 months. During this period, all eligible participants will be consecutively recruited to participate in the survey as a means to achieving maximum variation in clinical and sociodemographic characteristics and to facilitate comparison across key purposive sampling criteria [7]. Participants will be invited to complete the questionnaire 2 weeks after returning their stool self-sample pot, allowing a 3-week recall period from the date of contact with the NHS.

#### Survey Distribution

When a member of the public contacts a collaborating GP, they will receive information in their study pack about the patient experience survey. If they express an interest in finding out more about this survey, they will be asked to identify a preferred contact route by either receiving a self-completion questionnaire through the post or accessing a Web-based self-completion questionnaire.

If prospective participants choose to receive this information through the post, they will be sent a public survey information pack containing an introductory letter, an information sheet explaining the aims of the study and the processes involved in participating in the survey, a copy of the self-completion questionnaire, and a prepaid return envelope addressed to the Project Office at the University of Liverpool. If prospective participants prefer to access these details online, they will receive an email. The email will include an electronic link to a secure, Web-enabled system containing a study introduction page. If interested, prospective participants can then access 2 further links. The first link will open an information page, which will outline the aims of the study and processes involved in participating in the online survey. The second link will enable prospective participants to access and complete the electronic version of the self-completion questionnaire (through the PHE Select Survey facility [8]).

Participants will be given 2 weeks to complete and return/submit their questionnaire. Nonresponders will be prompted (through their preferred route) with 1 follow-up reminder. These will include, as appropriate, a follow-up letter containing a further copy of the questionnaire and a prepaid return envelope or a follow-up email with an electronic link to the online questionnaire. The use of reminders is generally endorsed in texts on survey methods [9].

### Summary of Outcome Measures

The outcome measures to be quantified within the research project are summarized in Table 1.

### Plan of Analyses

#### Anomaly Detection

The underlying statistical model for human case incidence will be a spatiotemporal Cox process [10] in which the rate of calls at location *x* and time *t* is modeled as *ρ(x,t)=λ(x)μ(t)R(x,t)*, where *λ(x)* and *μ(t)* describe the normal patterns of variation in the spatial and temporal dimensions, respectively, of the call rate, thereby taking account of the geographical distribution of the user population, seasonal variation in disease risk, and reporting artifacts such as day-of-the-week effects. The term *R(x,t)* is a stochastic process with expected value 1 and represents unforeseen, spatially and temporally localized variations in the underlying disease risk.

Model parameters will be estimated by likelihood-based methods and fed into algorithms that update the predictive distribution of the *unexpected* component *R(x,t)* automatically on receipt of each day’s incident call data. Results will be posted overnight in the form of maps showing localities, if any, where the data indicate a high probability that the current value of *R(x,t)* exceeds a specified threshold.

#### System Performance

The performance of the new surveillance system compared with routine sampling will be assessed by analyzing the outcome-based indicators and comparing time with detection, decision to act, and the size of outbreaks described using both traditional and new diagnostic systems (range, mean, and median number of cases), outbreak settings, modes of transmission identified, and vehicles identified. Each critical time point along the diagnostic and detection pathway from symptom onset to diagnosis and detection of a cluster or outbreak will be examined.

**Table 1:** Resource use and costs outcome measures to be quantified within the research program.

Outcome measures		How to measure
**Resource use and costs outcome measures**		
	Use of health care services	Resource use questionnaire (PES^a^) to patients
	Use of personal social services	Resource use questionnaire (PES) to patients
	Days absent from work or education	Resource use questionnaire (PES) to patients
	Other potential cost impacts	Resource use questionnaire (PES) to patients
	Use of secondary care services	Hospital Episode Statistics from NHS^b^ Digital
	Costs of technology	Interviews with Luminex (new technology) and time and motion studies at microbiology laboratories (existing technology)
Health outcome		EQ-5D-3L^c^ questionnaire administered at 2 time points: time point 1, during the acute illness; and time point 2, 2 weeks after return of the Acute Gastroenteritis Questionnaire
**System outcome measures^d^**		
	Time to detection of event	Laboratory records, date of AEGISS^e^ anomaly detection, date that Consultants in Communicable Disease Control initiate an investigation
	Compliance with sampling among people with symptoms	Laboratory records (number of samples requested) and GP^f^ records (number of samples submitted)
	Time to detection of a positive result	Laboratory records
	Numbers of false-positive and false-negative stool samples	Laboratory records
	Positive predictive value	Calculated from laboratory records using the formula: Σ true positives/Σ test outcome positives (ie, true positives + false positives)
	Diagnostic gap	Laboratory records: percentage of negative samples using either system
	Size of outbreaks detected	Outbreak investigation reports: range, mean, and median numbers of cases

^a^PES: Patient Experience Survey.

^b^NHS: National Health Service.

^c^EQ-5D-3L: EuroQol-5D-3L descriptive system.

^d^These will be captured for traditional methods and new diagnostic technology.

^e^AEGISS: Ascertainment and Enhancement of Gastrointestinal Surveillance and Statistics.

^f^GP: general practitioner.

#### Economic Modeling

The model structure will be based on a decision analysis in which the alternative options will be specified according to treatment pathways and strategies for public health intervention. The impact and scale of outbreaks will be modeled using agent-based models in which hypothetical cohorts are subject to an instantaneous rate of infection, which varies depending on the proportion of the population who are infected. This approach has several advantages over the traditional health economic models, which are restrictive in their predictive capabilities, and scenario testing. The model will be parameterized with point estimates and associated variances, derived from a purposive review of the published literature, from routinely collected data from PHE (both historical and contemporary), and from data generated during the research. These will include parameters relating to host-pathogen interactions; rate parameters that define the transition of patients among relevant states (eg, susceptible, diseased, and symptomatic and/or infectious); test characteristics, defined by sensitivity, specificity, and positive and negative predictive values; costs (associated with screening, patients’ use of NHS community, primary and secondary care services, treatments, and other investigations); and health outcomes (defined by health state utilities based on UK tariff scores assigned to each model state and mortality estimates).

#### Calculating and Judging Cost-Effectiveness

Expected costs and benefits will be estimated to calculate incremental cost-utility ratios (costs per QALY gained) for a range of scenarios, specified by infection type, clinical course, and public health response. Estimates of the incremental cost-effectiveness ratios (ICERs) will be compared with the £20,000 to £30,000 per QALY threshold of cost-effectiveness set by NICE, and a range of one-way sensitivity analyses will be conducted to assess the robustness of the analysis. These will be presented as a Tornado plot. Multivariate sensitivity analyses will be applied where interaction effects are suspected. The joint uncertainty in all parameter estimates will be propagated through the model by use of probabilistic sensitivity analysis and construction of cost-effectiveness acceptability curves that present the probability of clinical strategies being cost-effective, conditional on the chosen threshold for cost-effectiveness (representing the marginal value of health). Scenario analyses representing, for example, changes in service configuration will be conducted to estimate a range of ICERs for different circumstances.

#### Ethics

For the human case data, ethics permissions and approvals have been obtained from the following:

- National Research Ethics Service, REC reference: 15/NW/0233
- NHS Health Research Authority Confidential Advisory Group (CAG), CAG reference: 15/CAG/0131
- The Information Governance Toolkit, Department of Health and Social Care hosted by the Health and Social Care Information Centre (now NHS Digital), UoL reference: 8HN20, Lancaster University reference: EE133831-HAM-EAOPOCR
- NHS Research Management and Governance Committees, IRAS number: 173789
- University of Liverpool Ethics Sub-Committees, reference: UoL001111
- Honorary NHS contracts, research passports, and letters of access have been obtained for research staff working on the project as necessary.

## Results

The project commenced on April 1, 2013. Favorable approval was obtained from the Research Ethics Committee on June 15, 2015, and the first patient was recruited on October 13, 2015, with 1407 patients recruited and samples processed using traditional laboratory techniques as of March 2017.

## Discussion

This study investigates whether modern microbiological methods can be used to improve surveillance for GI disease while also examining the costs and limitations associated with the enhanced system. It compares the results obtained using traditional laboratory techniques with those obtained using modern sensitive molecular and genomic microbiology techniques. The strength of the study is the collaboration between lead public health partners and researchers in this field. However, there are a number of challenges in this study. For example, the plans for work are based on the assumption that implementation of the surveillance streams and their providers will continue in their current form for at least the period of study recruitment. Ethical permissions are granted under this proviso, but as with the provision of all health services, changes can occur rapidly. If there are changes to the study protocol, then these must be reflected in amendments to the ethics agreements, and the research governance including confidentiality agreements required for this to happen can take a significant amount of time to go through the review process, delaying the progression of data collection. Another challenge is that of recruiting a sufficiently large number of patients for analysis, to allow reasonable comparison of the results of the traditional and modern microbiological testing. This is particularly true, given that GPs often do not encourage patients to provide a stool sample unless they have had clinical GI symptoms for an extended period or unless they are in a high-risk group such as those who are young, old, or immunocompromised.

## Abbreviations

APHA: Animal and Plant Health Agency
CAG: Confidential Advisory Group
GI: gastrointestinal
GP: general practitioner
HPRU: Health Protection Research Unit
ICER: incremental cost-effectiveness ratios
NHS: National Health Service
NICE: National Institute for Health and Care Excellence
NIHR: National Institute for Health Research
PCR: polymerase chain reaction
PHE: Public Health England
QALY: quality-adjusted life-year
RCGP RSC NMN: Royal College of General Practitioners’ Research and Surveillance Centre National Monitoring Network
xTAG GPP: Luminex xTAG Gastrointestinal Pathogen Panel
